# A flow-through imaging system for automated measurement of ichthyoplankton

**DOI:** 10.1016/j.mex.2022.101773

**Published:** 2022-06-22

**Authors:** David R. Williamson, Trond Nordtug, Frode Leirvik, Bjarne Kvæstad, Bjørn Henrik Hansen, Martin Ludvigsen, Emlyn John Davies

**Affiliations:** a SINTEF Ocean, Climate and Environment, Brattørkaia 17C, 7010 Trondheim, Norway; b SINTEF Ocean, Fisheries and New Resources, Brattørkaia 17C, 7010 Trondheim, Norway; c Norwegian University of Science and Technology, Department of Marine Technology, 7491 Trondheim, Norway

**Keywords:** Microscopy, Flow cytometry, Computer vision, Machine learning, Automated biometry, Atlantic cod

## Abstract

Microscopic imaging and morphometric measurement of fish embryos and larvae is essential in environmental monitoring of fish populations and to evaluate larvae development in aquaculture. Traditional microscopy methods require time-consuming, repetitive work by human experts.

We present a method for fast imaging and analysis of millimetre-scale ichthyoplankton suspended in seawater. Our system can be easily built from common and off-the-shelf components and uses open-source software for image capture and analysis. Our system obtains images of similar quality to traditional microscopy, and biological measurements comparable to those by human experts, with minimal human interaction. This saves time and effort, while increasing the size of data sets obtained.

We demonstrate our approach with cod eggs and larvae, and present results showing biologically relevant endpoints including egg diameter, larval standard length, yolk volume and eye diameter, with comparison to similar measurements reported in the literature.

• High throughput, microscope-scale imaging of fish eggs and larvae

• Automated measurement of biologically relevant endpoints

• Easily built from off-the-shelf components and open-source software

## Graphical abstract

**Figure:**
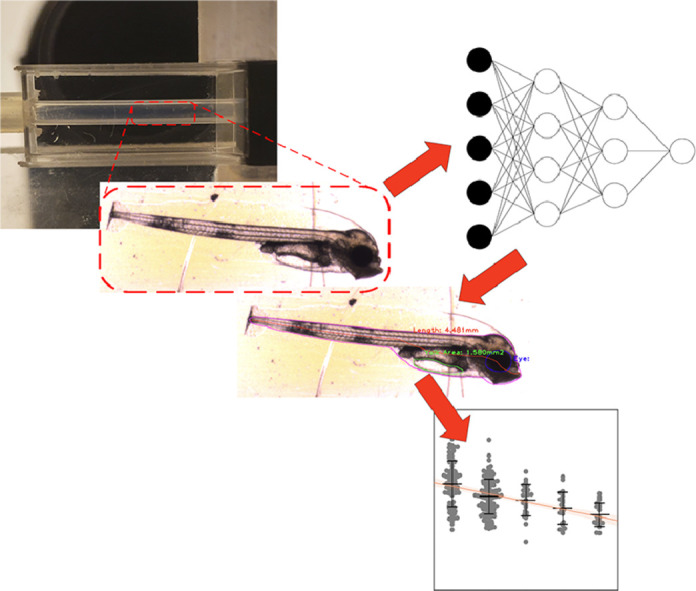

Specifications Table

**Table:** 

Subject Area:	Computer science
More specific subject area:	Computational biology, computer vision
Method name:	A flow-through imaging system for automated measurement of ichthyoplankton
Name and reference of original method:	An imaging in-flow system for automated analysis of marine microplankton [1] DOI: 10.33354/meps168285
Resource availability:	PySilCam software for image acquisition: https://github.com/SINTEF/PySilCam/ Fish annotator software: https://10.5281/zenodo.6565890 AutoMOMI: https://doi.org/10.5281/zenodo.5745208

## Method details

### Imaging system overview

**Fig. 1:**
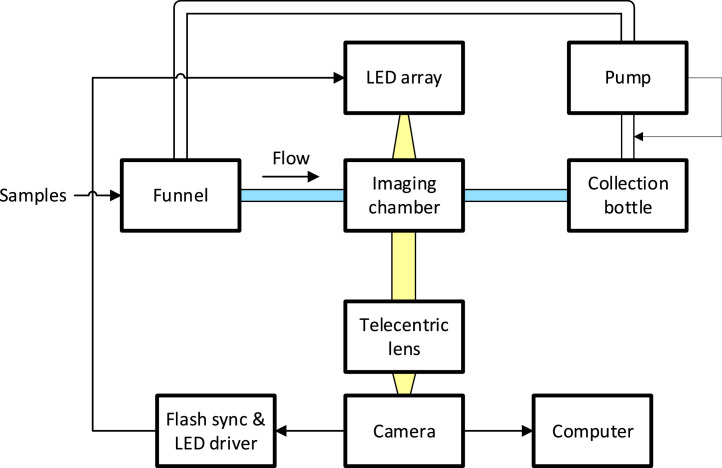
Flow diagram of experimental setup. Samples are introduced to the funnel and flow through the imaging chamber to the collection bottle, pulled by the vacuum from the pump. The pump introduces bubbles into the water in the funnel. Light from the LED array passes through the imaging chamber into the lens and camera. Images from the camera are saved to the computer, and the camera shutter triggers the LED array.

**Fig. 2:**
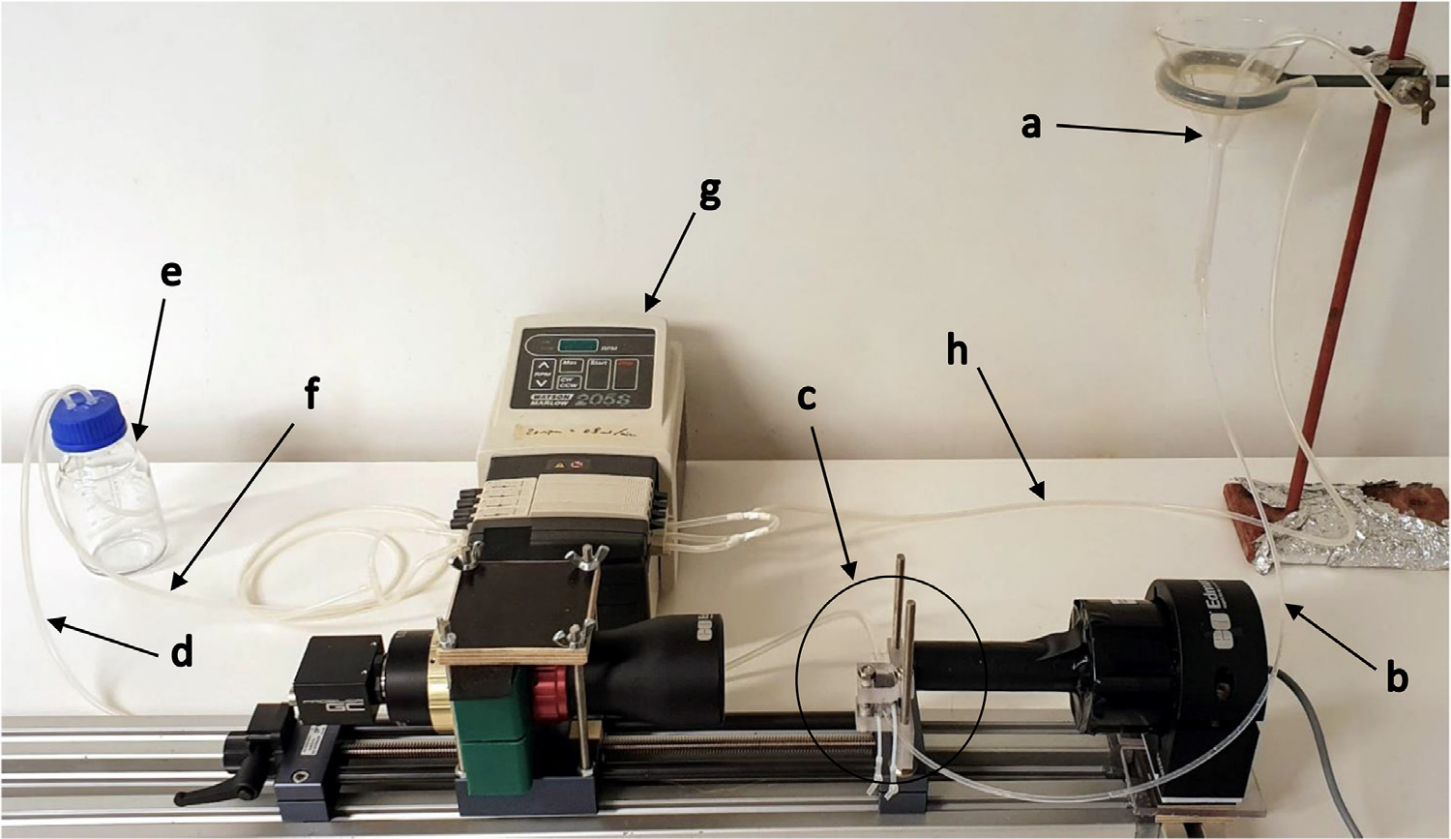
Setup showing the sample intake funnel (a) connected via a tube (b) to the imaging cell (c) connected via a tube (d) to the collection bottle (e), where a vacuum is drawn through a tube (f) by a peristaltic pump (g). A bubbler tube (h) runs from the pump into the funnel.

**Fig. 3:**
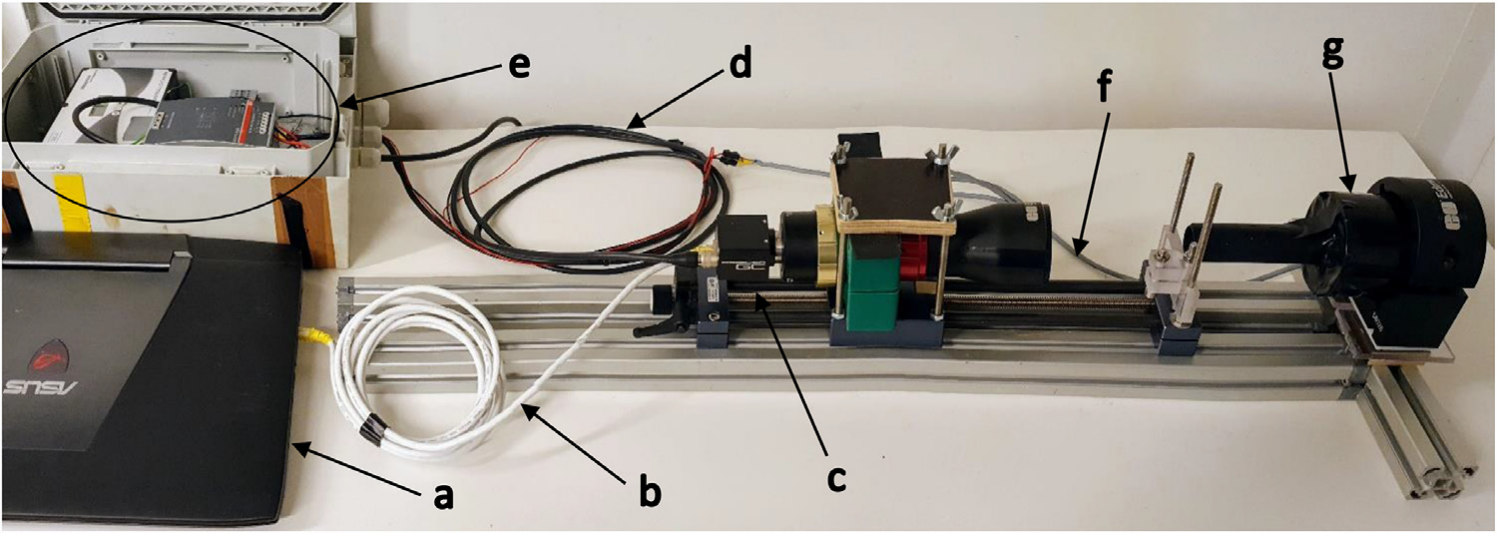
Setup showing electrical and data connections. The laptop (a) uses an ethernet cable (b) to power and control a camera (c). The camera sends a signal via (d) to the external LED controller (e), which uses a cable (f) to power and to trigger the strobe (g).

Seawater containing ichthyoplankton (e.g., fish eggs or larvae) in suspension is introduced through a funnel into a narrow tube. The tubing empties into a bottle through a hole drilled in the bottle lid. Another tube poking through the bottle lid connects to a peristaltic pump which pulls air from the bottle, creating a vacuum inside that draws water through the imaging system. A short section of tubing is imaged by the camera, illuminated by an LED array directing light through the tubing and its contents and into the camera lens. A diagram of these connections can be found in Fig. 1, and annotated photographs of the experimental setup can be seen in Fig. 2 and Fig. 3.

Images from the camera are recorded onto a computer. A subset of images is manually annotated with the outlines of features of interest, and these images and annotations comprise a training set for a neural network. Once trained, the network automatically selects and annotates features from the full set of images. Classical machine vision algorithms are then used to extract biologically relevant measurements of the fish eggs and larvae from these features.

### Flow

Seawater containing samples (fish eggs or larvae) to be imaged is introduced to a small, glass funnel mounted on a retort stand. Care must be taken to introduce the samples slowly and in portions to prevent the outflow of the funnel becoming clogged – drawing samples into a 10 ml syringe gives best control, or they can be poured from a small beaker. The end of this funnel is connected to approximately 600 mm of hard, clear perfluoroalkoxy alkane (PFA) 2 mm inner diameter (ID)/3 mm outer diameter (OD) tubing that runs into a cuvette mounted in front of the camera. After the cuvette, the tubing is connected to an approximately 900 mm length of soft, translucent Marprene 3 mm ID/5 mm OD tubing, terminating inside a 250 mL collection bottle through a hole in the bottle's lid. A peristaltic pump (Watson-Marlow, model 205S/CA4) is also connected to the collection bottle with approximately 1250 mm of Marprene tubing, again entering the bottle through a hole in the lid. When the pump is turned on, it creates a vacuum in the bottle, drawing first air and then seawater through the sample tube and into the collection bottle. The flow rate varies somewhat as the vacuum in the bottle builds, but reaches a rate of around 200 mm^3^ s^−1^ once the tubing is entirely filled with seawater. Also connected to the pump on the outflow side is approximately 1300 mm of Marprene tubing going into the sample funnel, which bubbles air through the water, helping to prevent samples clumping together. See Fig. 2 for an annotated photograph of the tubing layout.

### Imaging hardware

**Fig. 4:**
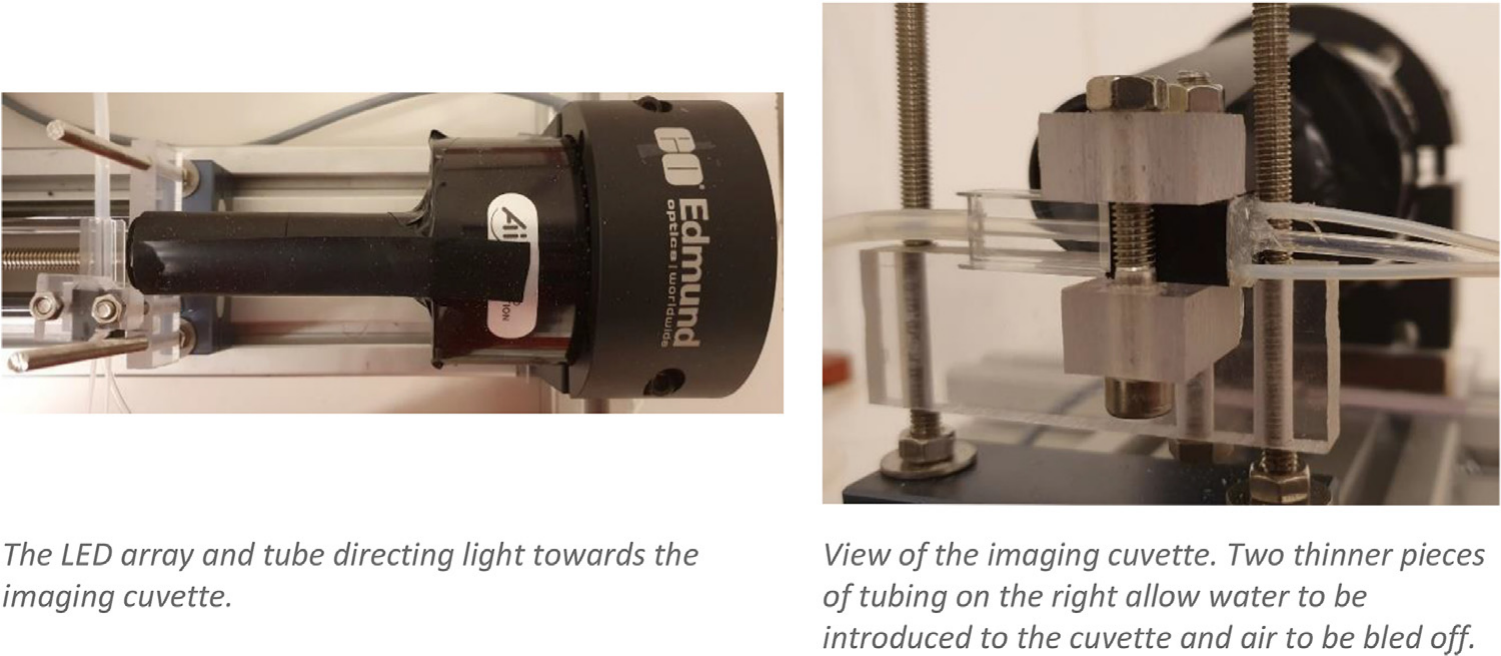
Detail of imaging setup.

The imaging components are mounted on a linear slide table (IGUS SHT-12) for alignment and to allow fine focus adjustment. Mounted at one end is a GigE Vision camera (2448 × 2048 pixels resolution, Allied Vision, Prosilica GC2450C, 2/3” sensor) with a telecentric lens (8.8 × 6.6 mm Field of View, 1x magnification, Edmund Optics #55–350). Telecentric lenses give an orthographic view of the subject, allowing the accurate measurement of in-focus samples regardless of their distance from the lens. Mounted 100 mm in front of the camera lens (inside the lens' approx. 3 mm useable depth of field) is the tubing section to be imaged. This section of tubing is enclosed in a 1 cm^3^ plastic cuvette with smaller tubes to allow it to be filled with water, made watertight with silicon sealant. This water-filled outer layer serves to reduce image distortion caused by the curved sides of the inner tubing. At the other end of the slide table is an LED array (Advanced Illumination, SL246), aimed directly into the camera lens. An opaque tube between the LED array and sample cuvette prevents light spillover around the edges of the imaging area, and a section of holographic diffusion film over the end of the opaque tube gives more even lighting (see Fig. 4).

### Image acquisition

The camera is connected over Ethernet to a laptop (ASUS, ROG Strix G751J) running the open source PySilCam image acquisition software [2]. Images were captured as single frames at a fixed 8 Hz in the camera native Bayer8 format, with an image size of 2448 × 750 pixels (cropped at capture from the 2448 × 2048 native camera resolution) and file size of around 1.8 MB per image. The LED array was synchronised to the frame capture: on frame capture start the camera sends a signal to the external trigger (Metaphase Technologies, Inc., ULC-2 Universal LED Controller), which fires the LED array. A strobe duration of 60 μs was used. Exposure time varied between 47 and 60 μs, depending on the age of the fish (older, more pigmented fish need a longer exposure time).

### Image analysis

**Fig. 5:**
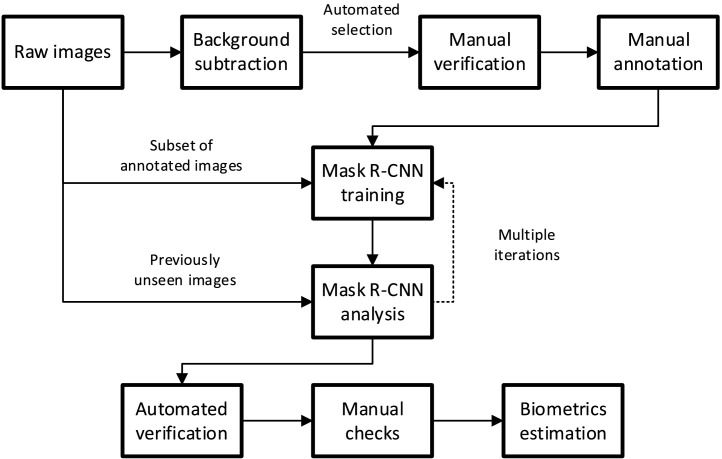
Image analysis pipeline. A portion of the raw images are annotated by hand and presented, with annotations, to the neural network for training. Following training the neural network attempts segmentation on unseen images, and the worst of these are annotated and added to the training set. The results receive additional post-processing, and biologically relevant endpoints are extracted.

Extracting useful measurements from the captured images requires first that frames containing eggs or larvae be identified. Relevant features (such as eyes or yolk sacs) are manually identified on a subset of images and used to train a neural network. The network then extracts these features from the complete set of images, and the results post-processed with classical machine vision techniques to correct or remove spurious results and calculate the sizes of the features in the image. This allows the estimation of biologically relevant metrics such as body and yolk sac volume, giving the growth rate and yolk sac utilization of the population over time. The steps in this image processing pipeline are shown in Fig. 5.

### Selection of candidate images

Most frames captured by the camera do not contain an egg or larva, though they may contain other particles such as air bubbles, dust specks or fibres. To identify frames of interest, a moving average background subtraction is first performed on each frame. The surrounding 50 frames are used to estimate a binary image of foreground and background pixels, where the foreground includes pixels significantly different from the average pixel value of those 50 frames. The area and bounding box of each “blob” of connected foreground pixels is calculated, and those that are too small to be an egg or fish are discarded. A portion of frames containing one or more large blobs of foreground images are selected and presented for manual checking.

### Manual feature annotation

**Fig. 6:**
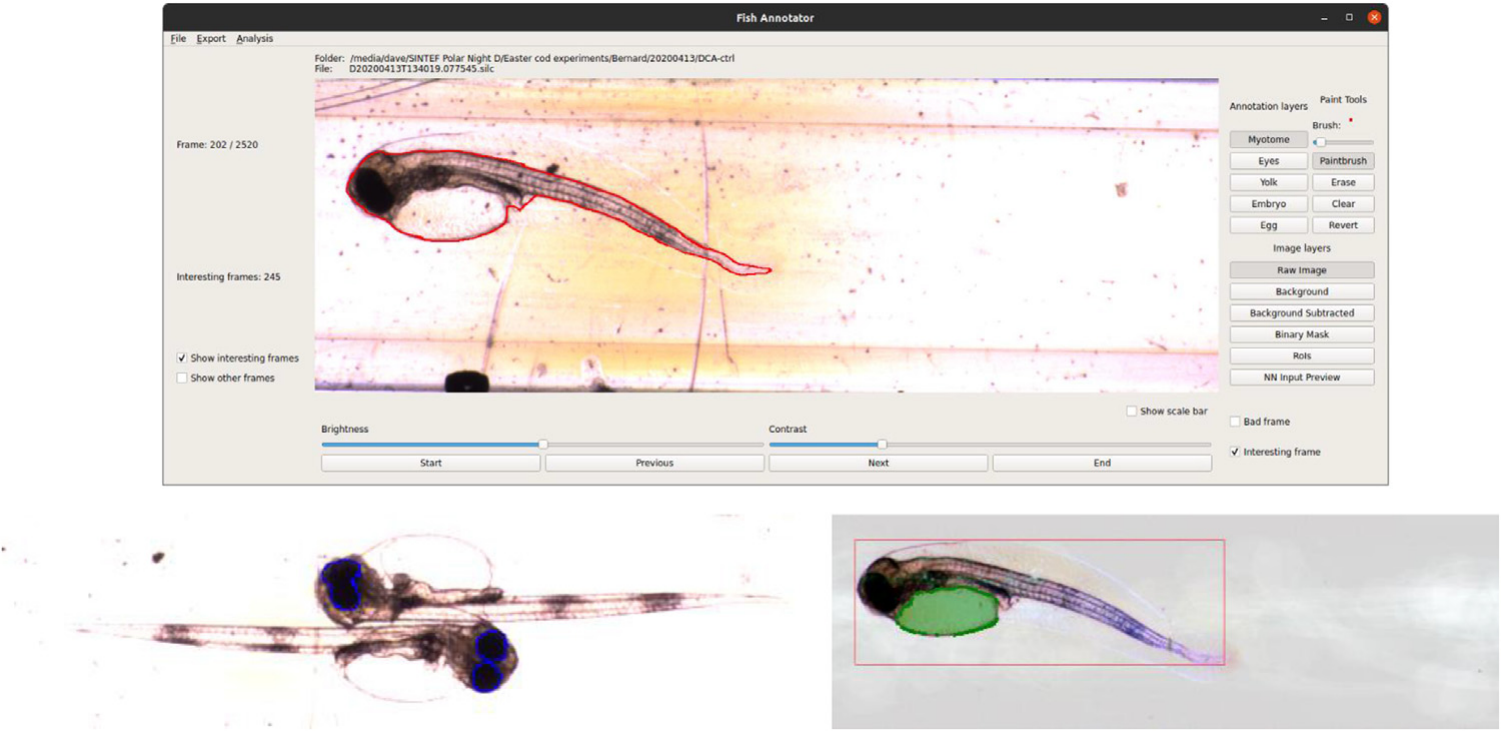
Examples of manual annotation. Top: larva body; bottom left: eyes; bottom right: yolk sac. These feature outlines (in red, blue, and green respectively) are drawn by hand and converted to a set of coordinates that are passed, along with the image, to the neural network for training. The lower two images (with the user interface cropped out) show use of image enhancements to make annotation easier: brightness and contrast adjustments, automated region of interest marking, background subtraction, and a preview of the feature points used in training the neural network.

From the automatically identified candidate frames, those not containing a fish egg or larva are discarded. The remainder are manually annotated using a graphics tablet and custom “Fish Annotator” software [3] to draw the outlines of visible features of interest. These features are: for eggs the egg shell, the embryo inside, and the non-yolk area of the egg; and for larvae the entire body, the eye(s) and the yolk sac (see Fig. 6).

Annotations are saved as lossless, 8-bit Portable Network Graphics (.png) files. The hand-drawn feature outlines are smoothed and parameterized as a polygon defined by a set of coordinates of evenly spaced points, and these coordinates are stored as comma-separated values (CSVs). The CSVs are used, along with the corresponding unmodified images and a pixel scaling parameter, as inputs for training the neural network. Annotated images are randomly assigned either to the training set (approximately 90%) or to the validation set (approximately 10%).

### Mask R-CNN

The neural network used is a modified implementation of Mask R-CNN [4], developed by Facebook AI Research (FAIR) [5]. The network takes as input images and annotations and produces segmentation masks for each feature class defined in training. A more comprehensive description and implementation details can be found in [6], and the open source AutoMOMI software used in training at [7], with the following differences for our imaging system:

• We train two networks, one for eggs and one for larvae.
• For larvae we have three classes: yolk, body, and eye.
• For eggs we have three classes: body, egg, and yolk.
• We set the hyperparameters max_gt_instances = 20 and mrcnn_mask_loss = 2

max_gt_instances limits the number of features detected in an image (by default 100). Although some images may contain several eggs, this will very rarely be more than 6 eggs x 3 classes = 18 features. Decreasing this limit reduces training time and false positives.

Increasing mrcnn_mask_loss (by default 1) adjusts the loss function, in effect making accurate segmentation masks more important to the neural network, and class identification and bounding box localisation less important. In our dataset, classes and bounding boxes are easily identified, but accurate segmentation is both difficult and important in accurately measuring our samples, so this encourages a network that will give good results in our application and may reduce training time.

Additionally, in [6] only one larva is present per image, so the post-processing of the network output produces only one mask per class. In our system several eggs or fish may appear in a single image, and the post-processing is modified to allow multiple masks per class.

**Fig. 7:**
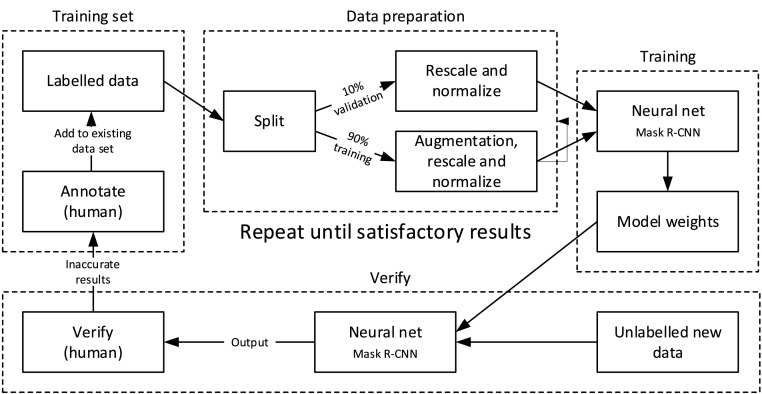
Mask R-CNN training. Adapted from [6], with permission.

The network is trained (see Fig. 7) for 900 iterations per epoch and TensorBoard^1^ used to monitor training, typically for 2-3000 epochs, taking several days using an Nvidia RTX2080 Ti graphics card. Epochs with low loss values are selected and their snapshot of model weights used to test segmentation on the validation set and a set of unannotated test images not previously seen by the network. The resulting segmentations are assessed by eye, and images with poor performance annotated manually and added to the training set. This training and annotation process is repeated through several iterations and the best performing set of model weights chosen to analyse the entire dataset.

**Fig. 8:**
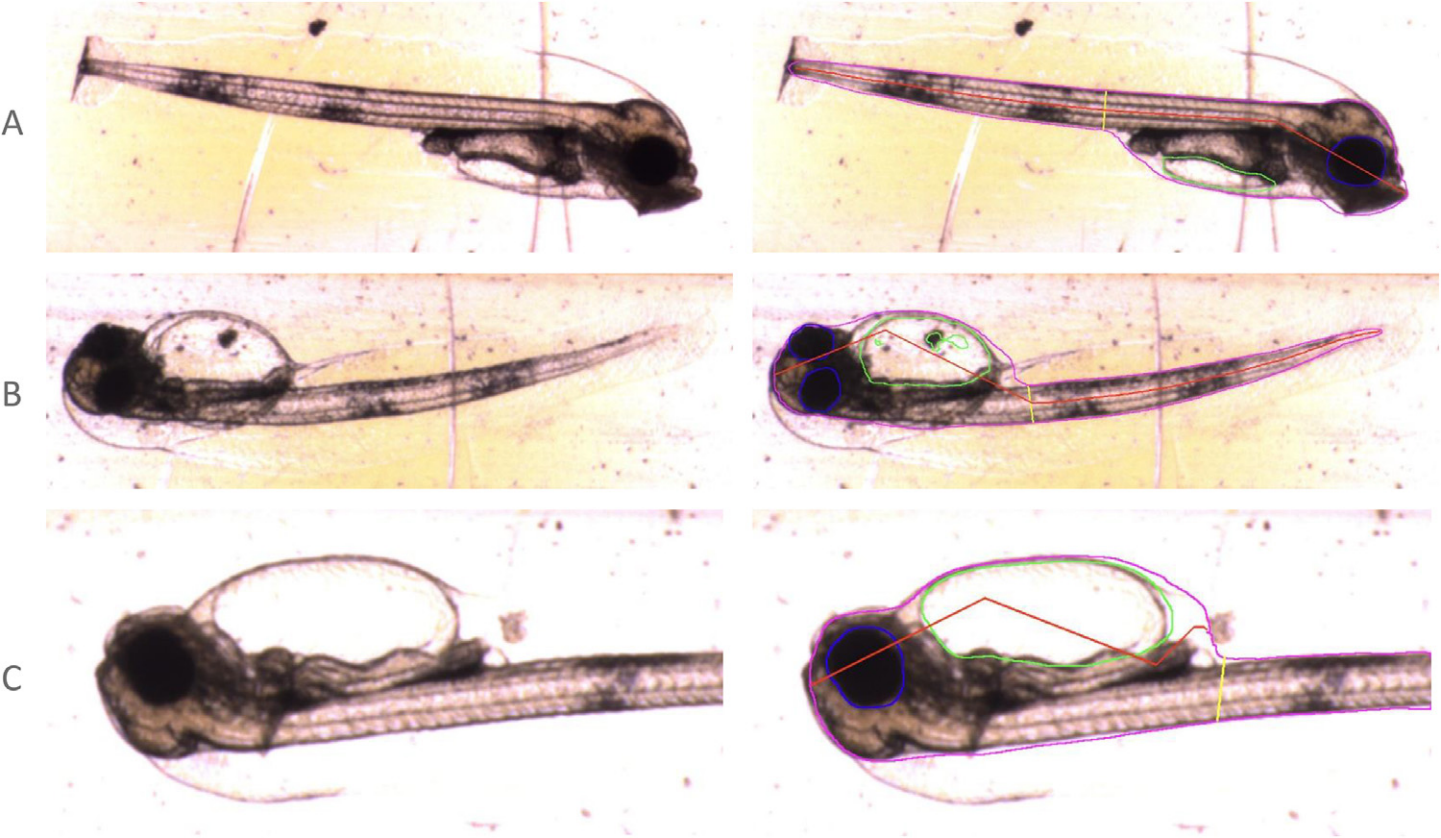
Examples of Mask R-CNN segmentations of larvae. The images presented to the neural network are shown on the left, segmentation results on the right (cropped to better show detail). Body, yolk sac and eyes are outlined in magenta, green and blue respectively. Red lines measure the myotome length and yellow its height. A shows an example of successful segmentation. B displays segmentation errors, such as the hole in the yolk sac and poor central body line in the inverted fish. C shows an example where the fish is partly off-frame, leading to inaccurate body measurements.

Examples of the segmentation results from Mask R-CNN on our dataset can be seen in Fig. 8, showing some errors in the neural network's attempts. The segmentation images were produced using code from [7].

### Classical machine vision post-processing

The neural network gives as output a list of labelled image regions for each image, corresponding to our feature classes. These segmentation masks are further processed with classical machine vision to remove results that are in error, and to extract biometric data. The following conditions are enforced on the Mask-RCNN segmentations of larvae:

• bounds of inner body parts such as eyes and yolk sacs are inside the bounds of the fish body
• fish have at most one body region
• fish have no more than two eyes
• fish have at most one yolk sac
• regions should have no internal “holes”, these are filled in
• body regions and body part bounds do not cross the image edges
• eyes and yolk sacs must be associated with a corresponding body

**Fig. 9:**
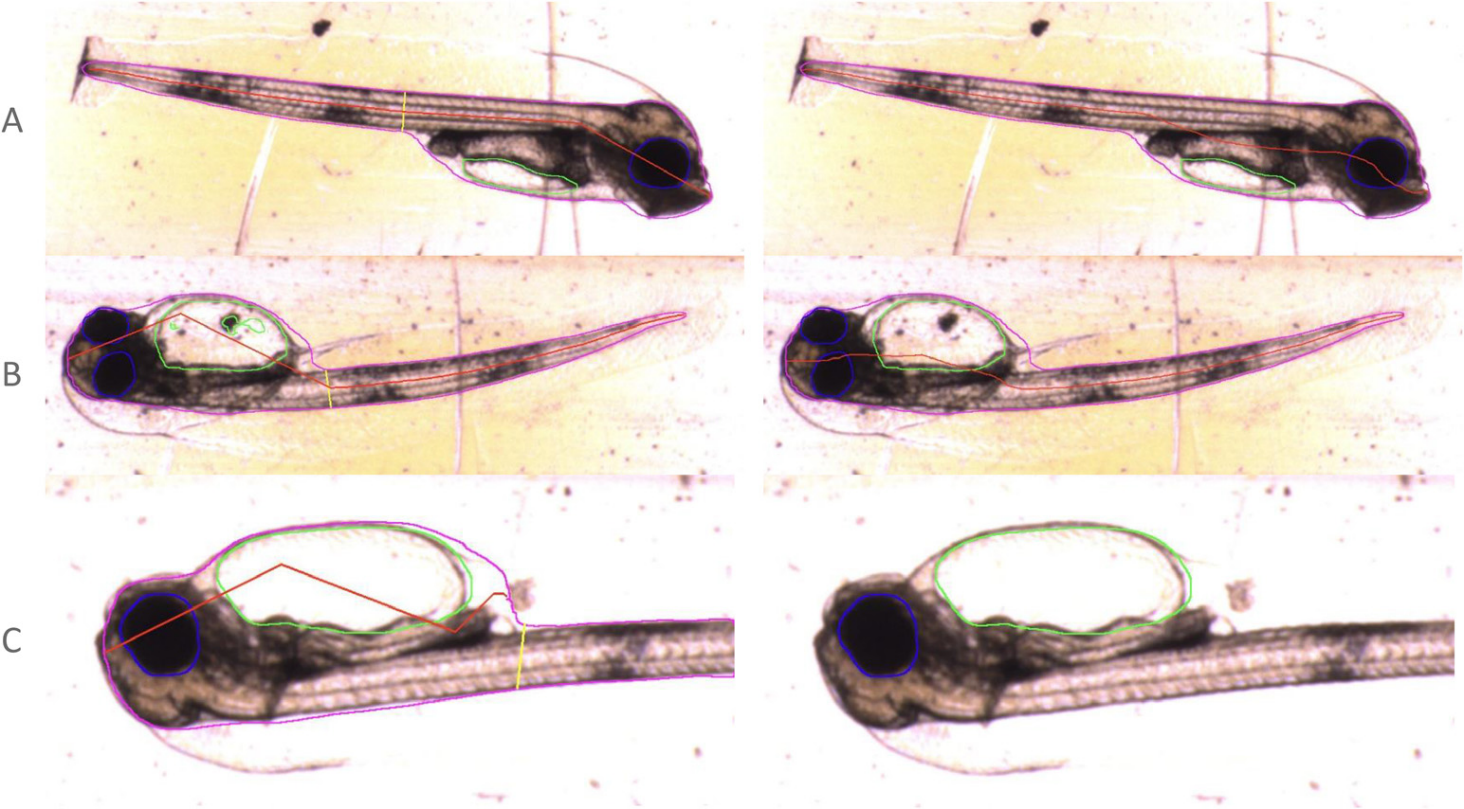
Examples of post-processed segmentations of larvae. Mask R-CNN segmentations are shown on the left, post-processing results on the right (cropped to better show detail). Outline colours as in Fig. 8. Note the improvements in the B, with the yolk sac filled and improved central body line. In C, the body segmentation intersecting the edge of the frame has been discarded, but eye and yolk sac measurements are retained.

Fig. 9 shows the same example images as in Fig. 8 following this post-processing.

**Fig. 10:**
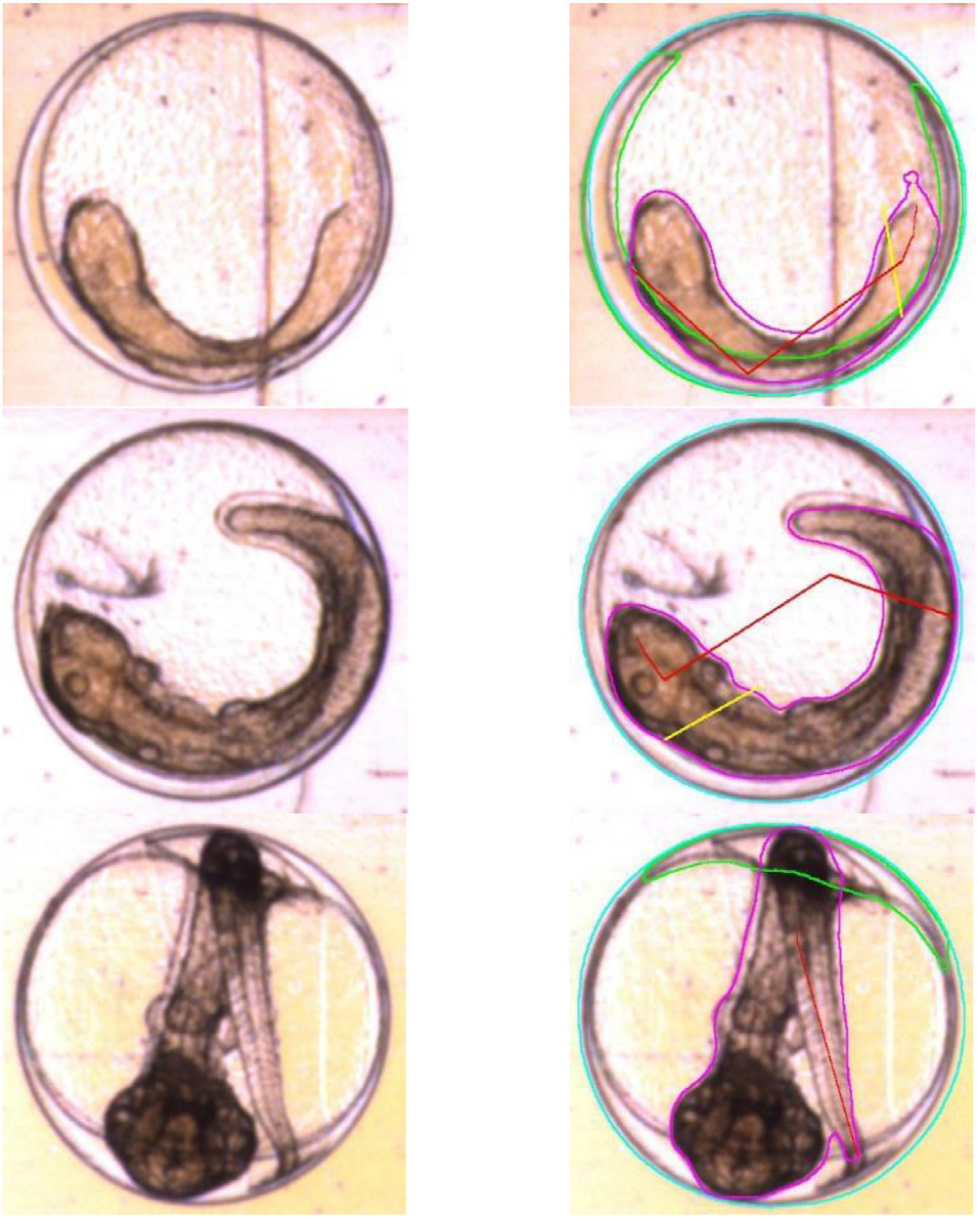
Examples of egg segmentation, cropped to show detail. Eggs are (top to bottom) 4, 7, and 9 days post-fertilisation. Original images on the left, Mask R-CNN segmentations on the right. Cyan lines show the shell of the egg, magenta the body of the embryo, and green the outlines of the yolk sac. Egg outlines tend to be accurate in these images, while body and yolk estimates are less reliable. Red and yellow lines are attempts at extracting myotome length and height, both of which fail badly on images of eggs.

After these checks and corrections, valid image regions are used to produce morphometrics. A centre line is extracted from the fish body, giving a standard length (SL) for each fish, and estimate the direction in which the fish is facing. The minor and major axes of the eyes and yolk sac are measured, as are the areas of body, eyes, and yolk sac. Measurements are converted from pixels to millimetres, and the volumes of these features estimated, since volume estimates are more widely used in the literature than simple lengths or areas.

### Larvae volume estimation

The total volume *V_L_* of an imaged larva is modelled as a cylinder. The projected area of the body *A_L_* is measured directly from the image segmentation, along with its standard length *L_L_*. Then,

$$V_{L}=\frac{A_{L}^{2}}{L_{L}}\;\frac{\pi }{4}$$

The structural volume of the larva, *V_S_*, is calculated as the total volume *V_L_* minus the volume of the yolk sac *V_Y_*, with *V_Y_* modelled as a prolate spheroid with the measured yolk length *L_Y_* and width *W_Y_*.

$$V_{Y}=\frac{4}{3}\pi {\left(W_{Y}/2\right)}^{2}\left(L_{Y}/2\right)$$

$$V_{S}=V_{L}-V_{Y}$$

The eye volume, *V_E_*, is also modelled as a prolate spheroid from the measured width and length, *W_E_* and *L_E_*. We use the width, *W_E_,* as the eye diameter in comparisons from the literature in the Method Validation section below.

$$V_{E}=\frac{4}{3}\pi {\left(W_{E}/2\right)}^{2}\left(L_{E}/2\right)$$

### Egg segmentation

Segmentations of eggs were found to be unreliable. While the outer shells of eggs were consistently detected, yolk areas were poorly segmented. Body regions were segmented more reliably, but it was not possible to estimate the orientation and pose of the body. This, along with the high curvature of bodies inside eggs leading to overlap in the image, made volume estimation of internal parts of the egg impossible to estimate. For this reason, body and yolk sac measurements in images of eggs are not used.

### Egg volume estimation

For cod eggs, a sphere is assumed for the total egg and a prolate spheroid for the yolk sac. Just after fertilisation the yolk sac is quite spherical, but its shape changes over development in the egg. In this study, since yolk sac measurements are unreliable, only the volume of the total egg is calculated. Major and minor axes (*L_E_* and *W_E_*) of the egg circle are measured directly from the image. These values are typically very similar, and their mean is taken to obtain an average egg diameter *D_E_*. This gives us the radius, *R_E_*, from which the egg volume, *V_E_*, is calculated.

$$D_{E}=\;\frac{L_{E}+W_{E}}{2};\;R_{E}=\frac{D_{E}}{2}$$

$$V_{E}=\;\frac{4}{3}\pi R_{E}^{3}$$

### Segmentation errors and misclassification

**Fig. 11:**
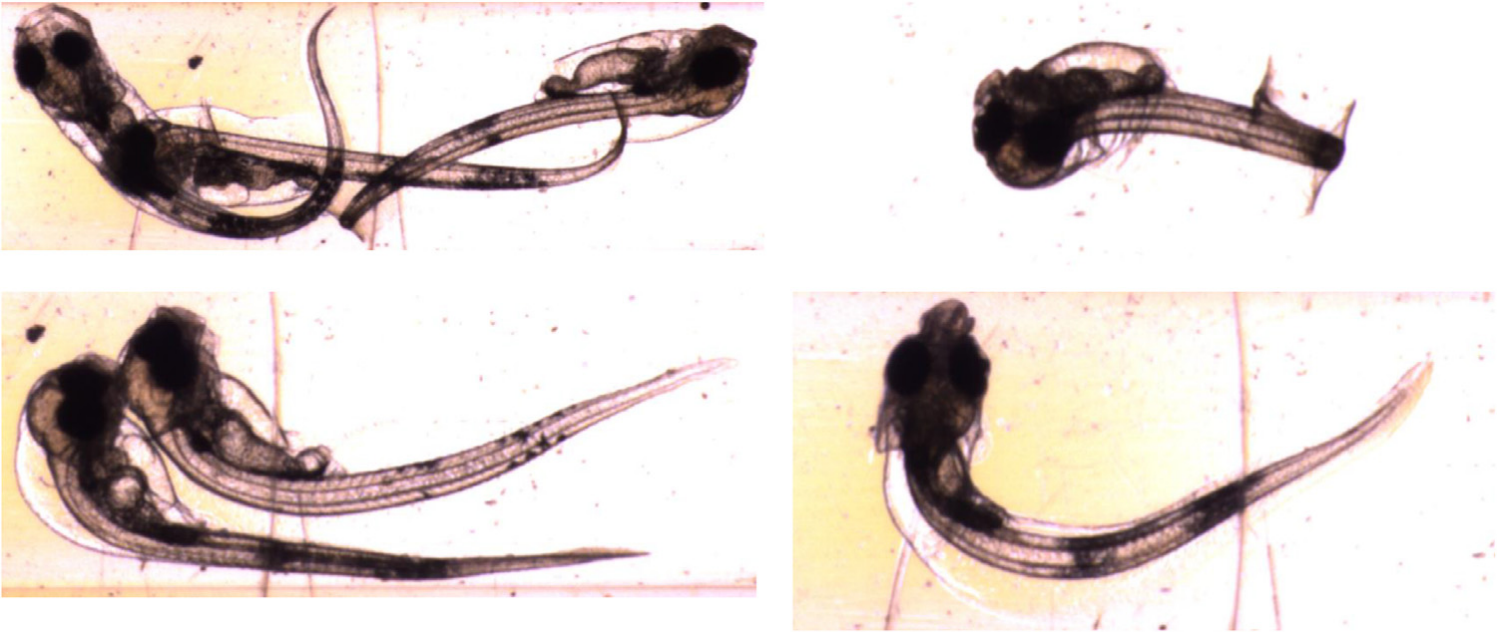
Examples of difficult to segment images, cropped to show detail. Clockwise from top-left: overlapping fish larvae; excessively curved body position; larva imaged from above; touching larvae.

Not all images captured are suitable for automated analysis with the current system. Larvae vary in pose and orientation, and only those facing the camera close to side-on can have their yolk sac and eye volumes reliably estimated. Fish can swim inside the imaging tube, occasionally leading to extremely curved body shapes that cannot be reliably measured. In some cases, fish or eggs appear partly out-of-frame – in these instances only features entirely within the image frame can be measured. Finally, some fish appear touching or overlapping one another, confusing the segmentation. In these difficult images yolk sac and eye measurements might still be possible, but automatic body segmentation typically fails. See Fig. 11 for examples of difficult to segment images.

## Method validation

### Motivation

Visual inspection to perform morphometric measurement and to assess deformations fish embryos and larvae is an important step in performing toxicology studies, but also to evaluate normal larvae development in aquaculture. The most common practise is to hand-pick individual eggs/larvae, image them under a microscope, and then perform measurements from these images. This is time-consuming and prone to human error and bias. Other work [6] has focused on automated measurement of manually posed and imaged fish larvae. We propose a method for fast, automated imaging of large numbers of ichthyoplankton in a laboratory setting, and modifications to the methods in [6] to accommodate our imaging system. Our method is non-destructive, allowing a population to be observed repeatedly over time, and uses low-cost equipment.

Our current system is intended for laboratory use, similar to the FlowCam fluorescence imaging system for microplankton from Fluid Imaging [1], or the REFLICS fish egg imager for ship-board use [8]. Future work will modify the system for *in-situ* imaging of ichthyoplankton, allowing fish eggs and larvae to be imaged in the ocean, and analysed automatically using the methods described here.

Much effort has gone into high-volume imaging and automated classification of non-ichthyous planktons, including the use of convolutional neural networks – see for instance [9] for a review including imaging methods and [10] for more recent work in classification. However, automated imaging and analysis of fish eggs and larvae has until now received relatively little attention, with no dedicated published data sets. Our system allows studies on early life stage fish to collect more data using less labour, and to quickly extract relevant growth metrics.

### Cod experiments

Atlantic Cod (*Gadus morhua*) eggs were supplied by NOFIMA on April 2nd, 2020, fertilized April 1st. Upon arrival, the temperature was 4 °C and oxygen was measured at 22.3 mg/L. The eggs were kept in a 200 L tank with flow-through filtered (1 µm) sea water at a temperature of 8.5 °C until 7 days post fertilization (dpf) when eggs were transferred borosilicate beakers (0.5 L) with increased seawater temperature (9–10 °C). Dead eggs and larvae were removed daily, and water renewed every 2–3 days. Imaging, as described above, started on April 4th (3 dpf) and ended on April 17th, approximately 5 days post hatch.

### Experimental results

**Fig. 12:**
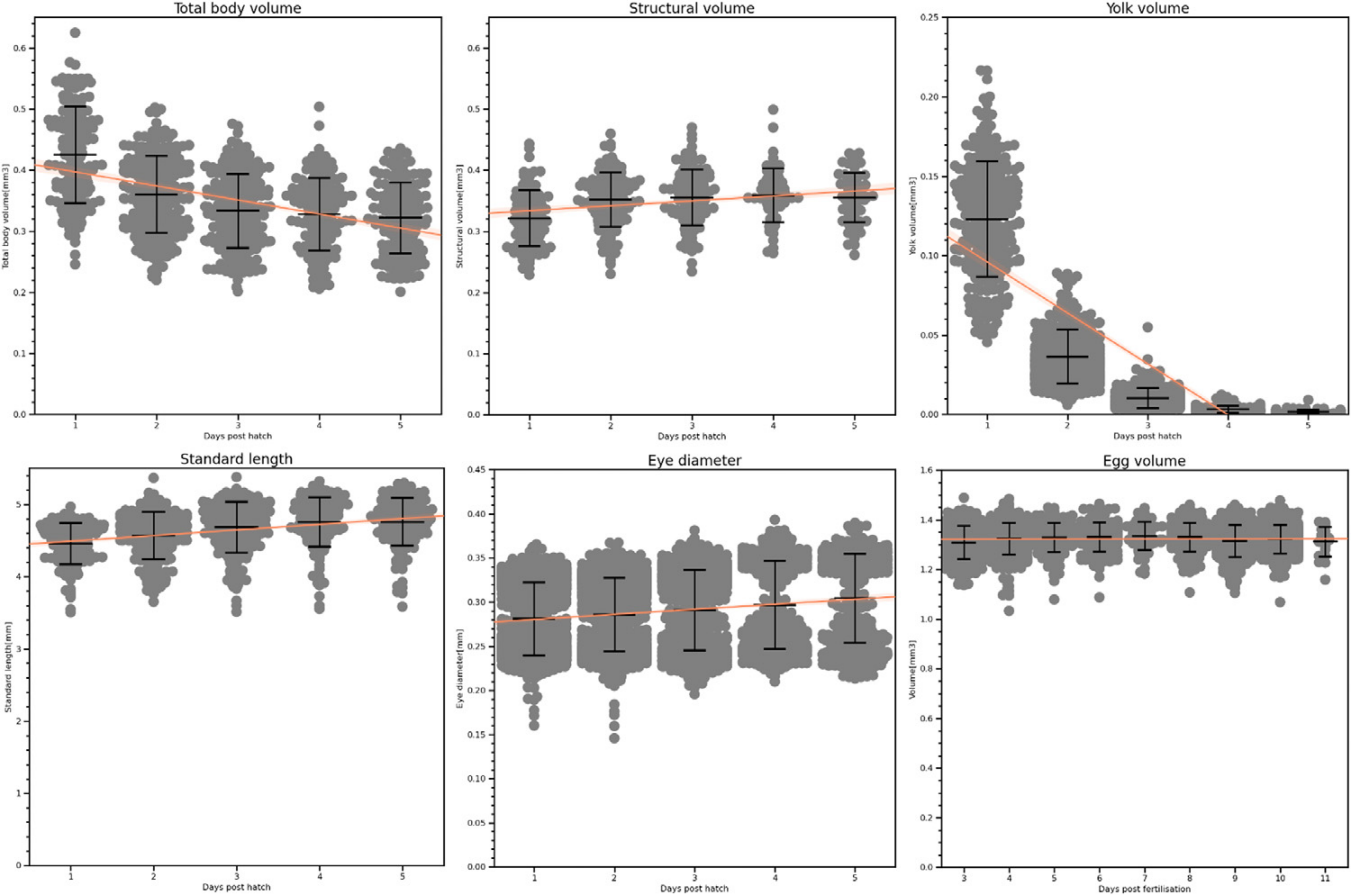
Endpoints for measurements of cod larvae and eggs. Swarm plots show individual data points with bars for the mean, upper and lower quartiles. Orange lines show linear regression fits.

We present results (Fig. 12) to demonstrate the ability of the automated system to collect reliable, biologically relevant data. One-dimensional metrics such as myotome length were measured directly, and volumes estimated from these measurements using the methods described above. As well as the automated checks described above, images resulting in extreme data points were checked manually and excluded if the segmentation was in error, for instance in cases such as those shown in Fig. 11.

## Discussion

We compare our results with other reported figures from the literature in Table 1.

**Table 1:** Comparison of our results with reported figures from the literature. Figures give the mean, plus or minus one standard deviation where available.

	Ours (13 dpf)	[11] (14 dpf)	[12] (16 dpf)	[13] (20 dpf)	[14]	[6] (20 dpf)
Egg diameter [mm]	1.36 ± 0.02		1.45 ± 0.02		1.1 – 1.4	
Standard length [mm]	4.48 ± 0.36	4.8 ± 0.1	4.03 ± 0.25	4.35 ± 2-3%		4.77 ± 0.13
Body area [mm^2^]	1.43 ± 0.14	1.42 ± 0.04				
Yolk area [mm^2^]	0.18 ± 0.05	0.08 ± 0.02				0.34 ± 0.03
Yolk volume [mm^3^]	0.03 ± 0.017		0.5			
Eye diameter [μm]	286 ± 4	284 ± 12				315 ± 15

The size of the eggs does not change significantly in the eight days observed. Eggs began to hatch on day 11 post-fertilisation, leading to fewer observations on this day. Egg diameter measurements fell within the bounds of reported values. We did not obtain other egg volume estimates from the literature, but the simple calculation from observed diameter to an estimate of a spherical volume can be expected to have a low error given the eggs' regular shape. Kjesbu *et al.* [14] report egg diameters between around 1.1 mm and 1.4 mm (estimated from plot) from three different female fish at a water temperature of 8 °C, but do not give the age of the eggs at measurement. Finn *et al.* [12] maintained eggs at 6 °C and say that their eggs were unusually large, with diameter 1.45 ± 0.02mm over the entire study and showing no significant change in volume during embryonic development.

In our results we see a steady growth in larvae length over the five days measured, and the standard lengths fall well within the range of other measurements from the literature. We anticipate that in some cases the length is underestimated, as swimming fish may be imaged with curled tails – this is not a problem in conventional microscopy where fish are typically immobilised and can be manipulated to lie straight.

Finn *et al.* [12] report a standard length of 4.03 ± 0.25 mm at 16 dpf, hatching occurring on day 15. Solberg and Tilseth [13] report hatching between 17 and 19 dpf in eggs reared at 5 °C, with a standard length of around 4.35 mm (estimated from plot) ± 2-3% (reported) at 20 dpf. Hansen *et al.* [11] give measurements for cod larvae in their control group at 14 days post fertilisation, and report an SL of 4.8 ± 0.1. The fish were maintained at a lower temperature (8.5 °C rather than our 9–10 °C), which will cause some difference in their rate of development. Kvæstad *et al.* [6] give an SL of 4.77 ± 0.13 for fish in their control group at 3 days post hatch (20 dpf), kept at 6 °C [15] – numbers from this study were calculated from raw data provided in personal correspondence with the authors.

The estimated total body volume of the fish decreases over time as yolk sacs are used up by the growing larvae. This is reflected also in the rapidly decreasing yolk sac volumes. In contrast, the structural volume, which excludes the yolk sac, increases as the fish grow. While we did not obtain such volume estimates from the literature, the body areas fell within reported values from [11] and [6]. Our yolk volume estimate differed significantly from that given in [12], reportedly calculated ”from the measured diameters using the formula for an ellipsoid” (the number in Table 1, Table 2 is estimated from a plot). It is unclear whether this is due to differences in calculation method or a genuine difference in size. It is possible that the lower temperature in that study led to slower yolk utilisation than in our fish.

**Fig. 13:**
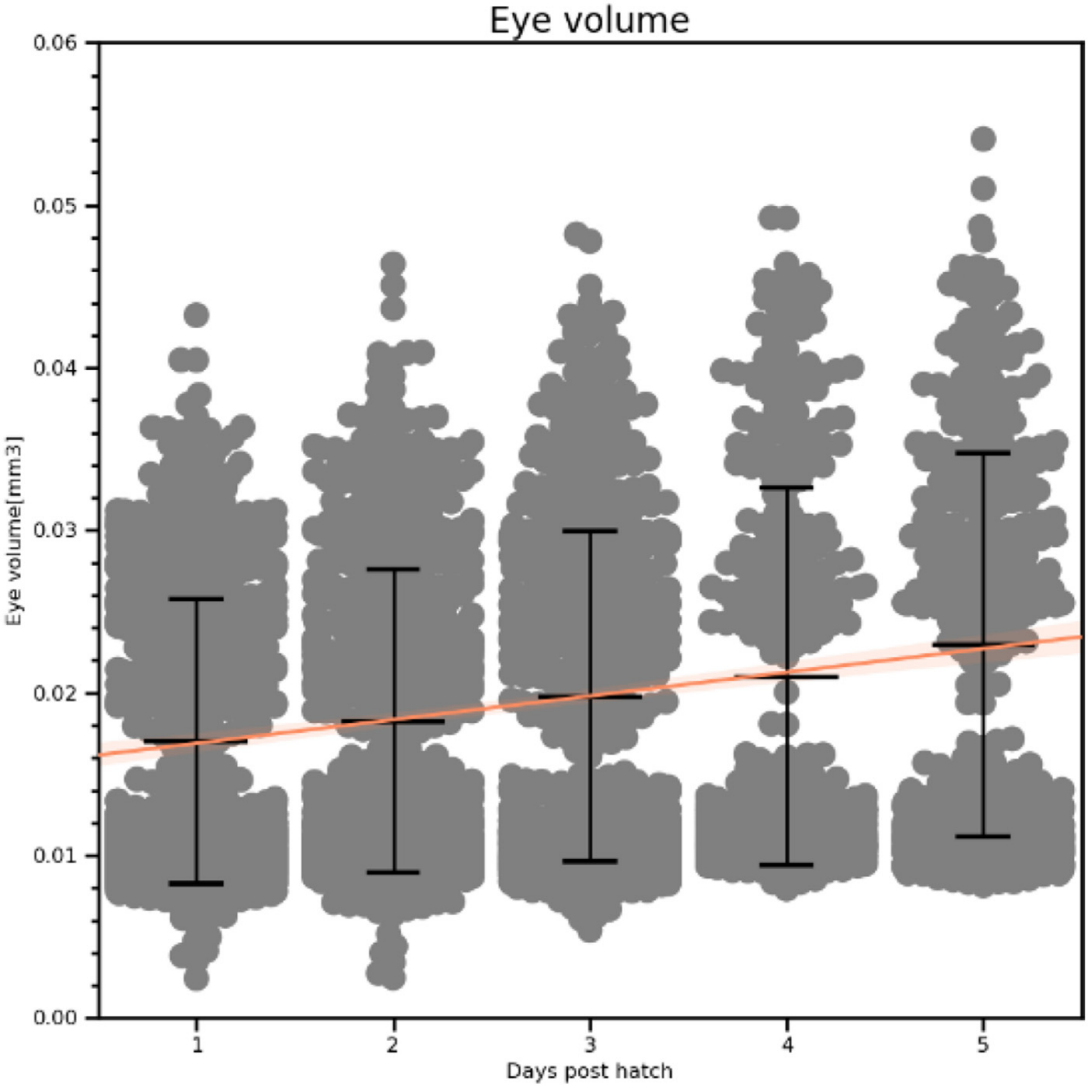
Eye volume estimates.

The eye diameter measurements show a bimodal distribution, which is clearer in the eye volume estimates (Fig. 13). This is due to a failure in segmentation: when a fish is angled such that the two eyes are partly overlapping from the camera's point of view, they are usually incorrectly treated as a single eye. The resulting segmentation in these cases has a figure-8 shape. This can lead to the eye diameter being underestimated as it measures the waist of the figure-8, and a higher estimated volume as the visible parts of both eyes increase the apparent size of the eye. While this did not lead to eye diameter measurements outside the ranges reported in [6] and [11], our eye volume estimates should be considered unreliable.

### Additional information: part selection

We include a list of the major parts used, along with a supplier and price at time of publication where available, to aid in costing and building the system. Standard, widely available components such as cables and fittings are not listed.

This system was built largely around components already available to us, and we hope readers take the parts listed as a guide rather than a prescription – we intend to demonstrate the system concept rather than advocate exact components. For instance, the GigE camera used could be replaced with a USB machine vision camera of similar resolution, or the LED array with a smaller light source. We especially note that the high-precision pump used in this system was conveniently available to us, but precise flow rate control is not required, and it could be replaced with a substantially less costly model.

We do recommend using the exact lens listed, however.

**Table 2:** List of major components.

Part	Supplier	Price (USD)
Allied Vision Prosilica GC2450C 2/3" CCD GigE Color Camera	Edmund Optics, #67-001	$3 220
GoldTL™ Telecentric Lens 1x magnification	Edmund Optics, #55-350	$1 765
Watson Marlow 205S/CA4 cartridge pump	Fisher Scientific, 14-284-101	$9 290
Metaphase Technologies ULC-2 universal LED controller	Metaphase Technologies, ULC-2	$500*
Advanced Illumination SL246 high intensity spot light	Edmund Optics, #18-602	$611
IGUS SHT-12 lead screw driven actuator	IGUS, SHT-12-SWM	$205

⁎ The manufacturer did not supply us with an up-to-date price, this is an estimate.

## Data Availability

Code is available at the links listed in the article. Training data has not been made publicly available, but data-sharing requests will be considered; please contact the corresponding author.

## Footnotes

1 https://www.tensorflow.org/tensorboard.
